# Supplementary material for: Metagenomic and taxonomic profiling of phyllosphere bacteria from Mangifera indica in response to urban air pollutants in Medellín, Colombia
Source: PLoS One. 2026 Apr 28;21(4):e0347959. doi: 10.1371/journal.pone.0347959 (PMC13124002; doi:10.1371/journal.pone.0347959)
Supplement: S1 File — S2 Fig. Rarefaction curves showing observed richness across samples at different sequencing depths. S3 Fig. Dot plot illustrating enriched aromatic compound degradation pathways in urban sites: a) DT S1, b) DT S2, c) SS S1, and d) SS S2. S1 Table. Summary of 16S rRNA gene amplicon sequencing output and quality-control processing statistics. S2 Table. Summary of shotgun metagenomics sequencing output and quality-control metrics. S3 Table. Assembly performance metrics derived from high-quality reads merged into scaffolds. S4 Table. Percentage of bin completeness and contamination in the samples (sampling period – urban sites) after bin refinement. S5 Table. Gene prediction outcomes generated using PROKKA and associated annotation statistics. S6 Table. Genes identified at S1 SS involved in catechol degradation via the ortho-intradiol cleavage pathway. S7 Table. Genes identified at S1 SS involved in catechol degradation via the meta-extradiol cleavage pathway. (ZIP) [file pone.0347959.s001.zip › Supporting_Information_13.04.2026/S1_table.docx]

**S1 table.** Summary of 16S rRNA gene amplicon sequencing output and quality-control processing statistics.

| **Sample** | **16S rRNA gene amplicon sequencing** | | | | | | **Quality Control** | |
| --- | --- | --- | --- | --- | --- | --- | --- | --- |
|  | **Total bases (pb)** | **Total reads** | **GC (%)** | **AT (%)** | **Q20 (%)** | **Q30 (%)** | **High-quality paired reads (%)** | **Bacterial reads (%)** |
| S1_DT_S1 | 306,213,320 | 1,017,320 | 56.5 | 43.5 | 88.8 | 78.0 | 58,265 (11.45) | 58,134 (11.43) |
| S1_DT_S2 | 247,865,072 | 823,472 | 56.2 | 43.8 | 89.0 | 78.1 | 41,529 (10.09) | 41,513 (10.09) |
| S1_DT_S3 | 351,236,298 | 1,166,898 | 57.0 | 43.0 | 89.0 | 78.3 | 76,278 (13.07) | 76,092 (13.04) |
| S1_SS_R1 | 274,238,090 | 911,090 | 56.5 | 43.5 | 90.3 | 80.8 | 41,331 (9.07) | 41,115 (9.02) |
| S1_SS_R2 | 324,544,220 | 1,078,220 | 56.5 | 43.5 | 91.0 | 81.7 | 52,155 (9.67) | 52,016 (9.64) |
| S1_SS_R3 | 277,539,458 | 922,058 | 56.3 | 43.7 | 91.0 | 81.8 | 42,752 (9.27) | 42,605 (9.24) |
| S2_DT_S1 | 289,779,322 | 962,722 | 57.2 | 42.8 | 85.2 | 73.4 | 47,000 (9.76) | 46,656 (9.69) |
| S2_DT_S2 | 324,237,802 | 1,077,202 | 56.5 | 43.5 | 88.8 | 78.6 | 59,523 (11.05) | 59,254 (11.00) |
| S2_DT_S3 | 316,333,542 | 1,050,942 | 57.0 | 43.0 | 88.1 | 77.6 | 68,549 (13.05) | 68,322 (13.01) |
| S2_SS_R1 | 288,992,508 | 960,108 | 57.3 | 42.7 | 86.1 | 75.2 | 54,972 (11.45) | 54,632 (11.34) |
| S2_SS_R2 | 252,289,772 | 838,172 | 56.9 | 43.1 | 86.8 | 76.0 | 46,362 (11.06) | 46,015 (10.98) |
| S2_SS_R3 | 210,822,206 | 700,406 | 56.8 | 43.2 | 86.6 | 75.5 | 32,206 (9.2) | 31,985 (9.14) |
