# Supplementary material for: Metagenomic and taxonomic profiling of phyllosphere bacteria from Mangifera indica in response to urban air pollutants in Medellín, Colombia
Source: PLoS One. 2026 Apr 28;21(4):e0347959. doi: 10.1371/journal.pone.0347959 (PMC13124002; doi:10.1371/journal.pone.0347959)
Supplement: S1 File — S2 Fig. Rarefaction curves showing observed richness across samples at different sequencing depths. S3 Fig. Dot plot illustrating enriched aromatic compound degradation pathways in urban sites: a) DT S1, b) DT S2, c) SS S1, and d) SS S2. S1 Table. Summary of 16S rRNA gene amplicon sequencing output and quality-control processing statistics. S2 Table. Summary of shotgun metagenomics sequencing output and quality-control metrics. S3 Table. Assembly performance metrics derived from high-quality reads merged into scaffolds. S4 Table. Percentage of bin completeness and contamination in the samples (sampling period – urban sites) after bin refinement. S5 Table. Gene prediction outcomes generated using PROKKA and associated annotation statistics. S6 Table. Genes identified at S1 SS involved in catechol degradation via the ortho-intradiol cleavage pathway. S7 Table. Genes identified at S1 SS involved in catechol degradation via the meta-extradiol cleavage pathway. (ZIP) [file pone.0347959.s001.zip › Supporting_Information_13.04.2026/S3_table.docx]

**S3 table.** Assembly performance metrics derived from high-quality reads merged into scaffolds.

| **Statistics** | **S1_DT_R1** | **S1_DT_R2** | **S1_DT_R3** | **S1_SS_R1** | **S1_SS_R2** | **S1_SS_R3** | **S2_DT_R1** | **S2_DT_R2** | **S2_DT_R3** | **S2_SS_R1** | **S2_SS_R2** | **S2_SS_R3** |
| --- | --- | --- | --- | --- | --- | --- | --- | --- | --- | --- | --- | --- |
| # scaffolds | 394109 | 306602 | 365139 | 212960 | 34022 | 9280 | 337460 | 237545 | 345029 | 338552 | 226185 | 287600 |
| # scaffolds (>= 0 bp) | 2221437 | 1871961 | 2196361 | 1277550 | 251491 | 146623 | 1878228 | 1456718 | 2085680 | 1850941 | 1582120 | 1792143 |
| # scaffolds (>= 1000 bp) | 160638 | 88957 | 127457 | 81955 | 8339 | 1824 | 117247 | 78666 | 123065 | 129938 | 73064 | 111279 |
| # scaffolds (>= 5000 bp) | 12762 | 7889 | 11323 | 14147 | 737 | 271 | 8477 | 7926 | 12919 | 13505 | 7743 | 10482 |
| # scaffolds (>= 10000 bp) | 3995 | 3805 | 4094 | 5698 | 441 | 197 | 2733 | 3067 | 3887 | 4407 | 2539 | 2887 |
| # scaffolds (>= 25000 bp) | 1113 | 848 | 934 | 1571 | 235 | 116 | 530 | 858 | 387 | 1015 | 451 | 465 |
| # scaffolds (>= 50000 bp) | 433 | 162 | 314 | 703 | 142 | 60 | 170 | 247 | 32 | 391 | 102 | 95 |
| Largest scaffold | 635045 | 1450957 | 1071359 | 993729 | 1136163 | 479365 | 331246 | 1051706 | 111920 | 791732 | 131544 | 188567 |
| Total length | 602150919 | 408235504 | 525277152 | 456936375 | 60139610 | 18453182 | 445204268 | 356242898 | 483962463 | 534330712 | 309746807 | 420652621 |
| Total length (>= 0 bp) | 1085832993 | 831937125 | 1018854806 | 738518258 | 119350325 | 54943748 | 862744827 | 687444306 | 953781505 | 940947700 | 674659318 | 825147490 |
| Total length (>= 1000 bp) | 438683940 | 258173659 | 360768219 | 366921246 | 42608622 | 13571239 | 292754822 | 247647345 | 332533211 | 389762363 | 205230669 | 299463707 |
| Total length (>= 5000 bp) | 164654524 | 125253689 | 154397040 | 236163587 | 30599645 | 11072997 | 98857535 | 119530452 | 125283727 | 172895805 | 85227909 | 106090208 |
| Total length (>= 10000 bp) | 105780077 | 96386299 | 105063349 | 177644446 | 28534406 | 10533157 | 60053926 | 86608504 | 63838233 | 110851392 | 49625503 | 54644714 |
| Total length (>= 25000 bp) | 63685109 | 51272743 | 58446120 | 116706378 | 25327614 | 9223082 | 27634235 | 53275835 | 13688242 | 61887593 | 18973441 | 19481906 |
| Total length (>= 50000 bp) | 40510685 | 28701046 | 37581659 | 86957303 | 22003473 | 7277377 | 15597388 | 32603539 | 2140256 | 40622869 | 7074549 | 6988225 |
| N50 | 1965 | 1448 | 1770 | 5467 | 5996 | 24316 | 1521 | 2026 | 1823 | 2199 | 1703 | 1955 |
| N90 | 657 | 607 | 628 | 698 | 627 | 609 | 617 | 620 | 615 | 649 | 608 | 632 |
| auN | 13018 | 36009 | 22386 | 45645 | 112176 | 80468 | 7988.6 | 36736 | 4818.5 | 16557 | 6556.3 | 5857.7 |
| L50 | 59506 | 43273 | 51413 | 12678 | 641 | 117 | 59426 | 27455 | 53984 | 43188 | 33389 | 45756 |
| L90 | 289767 | 232768 | 271751 | 135858 | 23312 | 5937 | 257532 | 173667 | 257790 | 245140 | 170135 | 212940 |
| GC (%) | 52.85 | 51 | 51.9 | 51.07 | 58.82 | 55.68 | 56.87 | 54.83 | 57.53 | 55.32 | 59.01 | 56.75 |
| # N's per 100 kbp | 1004.89 | 924.87 | 962.82 | 583.44 | 777.36 | 802.49 | 846.21 | 566.6 | 518.18 | 666.03 | 783.91 | 693.86 |
| # N's | 6050940 | 3775663 | 5057489 | 2665962 | 467499 | 148085 | 3767357 | 2018472 | 2507799 | 3558787 | 2428136 | 2918741 |
