# Supplementary material for: Metagenomic and taxonomic profiling of phyllosphere bacteria from Mangifera indica in response to urban air pollutants in Medellín, Colombia
Source: PLoS One. 2026 Apr 28;21(4):e0347959. doi: 10.1371/journal.pone.0347959 (PMC13124002; doi:10.1371/journal.pone.0347959)
Supplement: Attachment — Submitted filename: Response to Reviewers_PLOS ONE_14.12.2025.docx [file pone.0347959.s002.docx]

**Response to Journal Requirements — PLOS ONE**

**Manuscript ID:** PONE-D-25-50660
**Title:** *Metagenomic and taxonomic profiling of phyllosphere bacteria from Mangifera indica in response to urban air pollutants in Medellín, Colombia.*

Dear Academic Editor,

We thank you for the opportunity to revise our manuscript and for the detailed guidance provided. Below, we address each journal requirement point-by-point and describe the corresponding actions taken in the revised submission.

1. Please ensure that your manuscript meets PLOS ONE’s style requirements, including those for file naming. The PLOS ONE style templates can be found at

[https://journals.plos.org/plosone/s/file?id=wjVg/PLOSOne_formatting_sample_main_body.pdf](https://track.editorialmanager.com/CL0/https:%2F%2Fjournals.plos.org%2Fplosone%2Fs%2Ffile%3Fid=wjVg%2FPLOSOne_formatting_sample_main_body.pdf/1/010f019a32d6a5cc-3f3b2389-07e1-4830-b973-caa9fc88b0c5-000000/nllUJNIMVLwZwDUauXwgK6gW4mdEEtEOLEmTL5IOzq8=235) and

[https://journals.plos.org/plosone/s/file?id=ba62/PLOSOne_formatting_sample_title_authors_affiliations.pdf](https://track.editorialmanager.com/CL0/https:%2F%2Fjournals.plos.org%2Fplosone%2Fs%2Ffile%3Fid=ba62%2FPLOSOne_formatting_sample_title_authors_affiliations.pdf/1/010f019a32d6a5cc-3f3b2389-07e1-4830-b973-caa9fc88b0c5-000000/ibEk4rJEh-JJPXV25exdnJa1N9LqwlrTeOgRY90bxbM=235).

**Response:** This issue has been fully resolved. The manuscript has been reformatted according to the PLOS ONE templates for the main text and title page. All files now follow the appropriate naming conventions required by the journal.

2. Please note that PLOS One has specific guidelines on code sharing for submissions in which author-generated code underpins the findings in the manuscript. In these cases, we expect all author-generated code to be made available without restrictions upon publication of the work. Please review our guidelines at <https://journals.plos.org/plosone/s/materials-and-software-sharing#loc-sharing-code> and ensure that your code is shared in a way that follows best practice and facilitates reproducibility and reuse.

**Response:**

All code and data generated for this study have been deposited in a public repository in accordance with PLOS ONE’s data availability requirements. The dataset is available through the NCBI Sequence Read Archive under the following accession link: <https://www.ncbi.nlm.nih.gov/sra/PRJNA1269120>.

During the submission process, the NCBI platform required the specification of a public release date for the sequences, which was initially set to June 1, 2027. We have already contacted NCBI to request a modification of this date so that the data can be made publicly available as soon as possible. We will ensure that access is updated promptly and fully complies with the journal’s open data policies.

3. In your Methods section, please provide additional information regarding the permits you obtained for the work. Please ensure you have included the full name of the authority that approved the field site access and, if no permits were required, a brief statement explaining why.

**Response:** The manuscript has been updated to include the full permit information. We added the following text to the Methods section:

“Fieldwork was conducted under the General Permit for the Collection of Specimens of Wild Species of Biological Diversity for Non-Commercial Scientific Research, issued by the National Authority of Environmental Licenses (Autoridad Nacional de Licencias Ambientales, ANLA) of the Republic of Colombia through Resolution 1566 of July 24, 2024. This permit authorizes the collection of biological material within the national territory exclusively for scientific purposes and without commercial intent. All sampling and handling activities were covered under this general permit; therefore, no additional permits from regional or local authorities were required.”

4. We note that Figures S1 in your submission contain [map/satellite] images which may be copyrighted. All PLOS content is published under the Creative Commons Attribution License (CC BY 4.0), which means that the manuscript, images, and Supporting Information files will be freely available online, and any third party is permitted to access, download, copy, distribute, and use these materials in any way, even commercially, with proper attribution. For these reasons, we cannot publish previously copyrighted maps or satellite images created using proprietary data, such as Google software (Google Maps, Street View, and Earth). For more information, see our copyright guidelines: <http://journals.plos.org/plosone/s/licenses-and-copyright>.

We require you to either (1) present written permission from the copyright holder to publish these figures specifically under the CC BY 4.0 license, or (2) remove the figures from your submission:

 a. You may seek permission from the original copyright holder of Figures S1 to publish the content specifically under the CC BY 4.0 license.

We recommend that you contact the original copyright holder with the Content Permission Form (<http://journals.plos.org/plosone/s/file?id=7c09/content-permission-form.pdf>) and the following text:

“I request permission for the open-access journal PLOS ONE to publish XXX under the Creative Commons Attribution License (CCAL) CC BY 4.0 (<http://creativecommons.org/licenses/by/4.0/>). Please be aware that this license allows unrestricted use and distribution, even commercially, by third parties. Please reply and provide explicit written permission to publish XXX under a CC BY license and complete the attached form.”

Please upload the completed Content Permission Form or other proof of granted permissions as an "Other" file with your submission.

In the figure caption of the copyrighted figure, please include the following text: “Reprinted from [ref] under a CC BY license, with permission from [name of publisher], original copyright [original copyright year].”

 b. If you are unable to obtain permission from the original copyright holder to publish these figures under the CC BY 4.0 license or if the copyright holder’s requirements are incompatible with the CC BY 4.0 license, please either i) remove the figure or ii) supply a replacement figure that complies with the CC BY 4.0 license. Please check copyright information on all replacement figures and update the figure caption with source information. If applicable, please specify in the figure caption text when a figure is similar but not identical to the original image and is therefore for illustrative purposes only.

The following resources for replacing copyrighted map figures may be helpful:

USGS National Map Viewer (public domain): <http://viewer.nationalmap.gov/viewer/>

The Gateway to Astronaut Photography of Earth (public domain): <http://eol.jsc.nasa.gov/sseop/clickmap/>

Maps at the CIA (public domain): <https://www.cia.gov/library/publications/the-world-factbook/index.html> and <https://www.cia.gov/library/publications/cia-maps-publications/index.html>

NASA Earth Observatory (public domain): <http://earthobservatory.nasa.gov/>

Landsat: <http://landsat.visibleearth.nasa.gov/>

USGS EROS (Earth Resources Observatory and Science (EROS) Center) (public domain): [http://eros.usgs.gov/#](http://eros.usgs.gov/)

Natural Earth (public domain): <http://www.naturalearthdata.com/>

**Response:** We agree with the editor’s assessment. The original satellite-based map has been removed and replaced with an original sketch created by the authors that illustrates the location of the sampling sites. The new figure fully complies with CC BY 4.0 requirements, and the figure caption has been updated accordingly.

5. If the reviewer comments include a recommendation to cite specific previously published works, please review and evaluate these publications to determine whether they are relevant and should be cited. There is no requirement to cite these works unless the editor has indicated otherwise.

**Response:** All suggested publications were reviewed in detail. Relevant works have been incorporated into the revised Introduction and Discussion sections when appropriate. Citations were added only when they contributed meaningfully to the scientific context and aligned with the recommendations from the editorial team.

Reviewers' comments:

Reviewer's Responses to Questions

**Comments to the Author**

1. Is the manuscript technically sound, and do the data support the conclusions?

The manuscript must describe a technically sound piece of scientific research with data that supports the conclusions. Experiments must have been conducted rigorously, with appropriate controls, replication, and sample sizes. The conclusions must be drawn appropriately based on the data presented.

Reviewer #1: Yes

Reviewer #2: Partly

Reviewer #3: Yes

Reviewer #4: Yes

2. Has the statistical analysis been performed appropriately and rigorously?

Reviewer #1: Yes

Reviewer #2: Yes

Reviewer #3: Yes

Reviewer #4: Yes

3. Have the authors made all data underlying the findings in their manuscript fully available?

The [PLOS Data policy](http://track.editorialmanager.com/CL0/http:%2F%2Fwww.plosone.org%2Fstatic%2Fpolicies.action%23sharing/1/010f019a32d6a5cc-3f3b2389-07e1-4830-b973-caa9fc88b0c5-000000/aWwuK0DKdStRwnQeFyAWpkZS1XGd_3tjSYLZOyyGqDs=235) requires authors to make all data underlying the findings described in their manuscript fully available without restriction, with rare exception (please refer to the Data Availability Statement in the manuscript PDF file). The data should be provided as part of the manuscript or its supporting information, or deposited to a public repository. For example, in addition to summary statistics, the data points behind means, medians and variance measures should be available. If there are restrictions on publicly sharing data—e.g. participant privacy or use of data from a third party—those must be specified.

Reviewer #1: Yes

Reviewer #2: No

Reviewer #3: Yes

Reviewer #4: Yes

4. Is the manuscript presented in an intelligible fashion and written in standard English?

PLOS ONE does not copyedit accepted manuscripts, so the language in submitted articles must be clear, correct, and unambiguous. Any typographical or grammatical errors should be corrected at revision, so please note any specific errors here.

Reviewer #1: Yes

Reviewer #2: Yes

Reviewer #3: Yes

Reviewer #4: Yes

5. Review Comments to the Author

Please use the space provided to explain your answers to the questions above. You may also include additional comments for the author, including concerns about dual publication, research ethics, or publication ethics. (Please upload your review as an attachment if it exceeds 20,000 characters)

Reviewer #1:

I commend the authors for conducting a novel and technically ambitious study exploring how urban air pollution influences the phyllosphere microbiome of Mangifera indica. The integration of both 16S rRNA amplicon and shotgun metagenomic sequencing provides valuable ecological and functional insights. The manuscript is generally well structured, and the data are potentially of broad relevance to microbial ecology, environmental health, and urban sustainability research.

That said, several methodological and interpretative aspects require clarification or expansion to strengthen the robustness and reproducibility of the findings. Specifically, greater detail is needed on the sampling design, contamination controls, MAG quality, and statistical reporting. Additionally, the language can be refined for conciseness and clarity, and the conclusions should more cautiously reflect the evidence presented.

Addressing these issues through revision will substantially improve the scientific soundness and readability of the paper. I appreciate the authors’ efforts and hope these comments are helpful toward enhancing the manuscript’s quality.

**Response:** We thank Reviewers for the positive and encouraging assessment of our work. We appreciate the recognition of the novelty and technical integration of both 16S rRNA and shotgun metagenomic approaches. We also thank the reviewer for the constructive suggestions, which have helped substantially improve the scientific rigor and clarity of the manuscript.

As requested, we have addressed all methodological and interpretative concerns by:

1. Expanding the description of the **sampling design**, including selection criteria, replication structure, and site characterization.
2. Providing a clearer explanation of **contamination control measures**, both during sampling and laboratory processing.
3. Reporting explicit **MAG quality metrics** (completeness, contamination, N50) following MIMAG standards, and adding a supplementary table summarizing these metrics.
4. Improving **statistical reporting**, adding exact p-values, clarifying model structure, and specifying parameters used in multivariate analyses.
5. Refining the **language throughout the manuscript** to improve clarity and conciseness.
6. Revising the **Conclusions section** to ensure statements align strictly with the evidence and to avoid potential overinterpretation.

All these revisions strengthen the robustness and interpretability of the findings, and we believe they address the reviewer’s concerns thoroughly.

**Major Comments**

Sampling design and replication:

The manuscript should explicitly state whether the triplicate samples collected per site and time represent true biological replicates (distinct trees) or technical replicates from the same individual. This distinction is critical for assessing statistical independence and ecological inference.

**Response:** We thank the reviewer for raising this important point. In our study, the three samples collected per site and time point represent **technical replicates obtained from the same individual tree,** rather than biological replicates from different individuals. Our sampling strategy was intentionally designed to **track within-tree temporal dynamics** while minimizing between-tree heterogeneity, which is known to be substantial in phyllosphere systems.

This approach is consistent with previous phyllosphere and plant-microbiome studies that have emphasized the value of **repeated sampling of the same host individual** to capture temporal variation and reduce confounding effects caused by inter-individual variability (Redford et al., 2010*; Laforest-Lapointe et al., 2016**; Copeland et al., 2015***). Such designs have been shown to provide robust insights into microbiome temporal stability and host-specific patterns, even when biological replication at each time point is limited.

We acknowledge, as the reviewer notes, that this design does not provide independent biological replicates at each time point. To avoid pseudo-replication and ensure appropriate inference, we have now:

1. Explicitly stated in the Methods that the triplicate samples are **technical replicates** from the same tree.
2. Clarified that resulting ecological inferences represent **within-individual temporal responses** and not population-level generalization across trees.
3. Treated longitudinal measurements as **repeated measures** in the statistical analyses, following best practices for non-independent microbiome samples (Ladau & Eloe-Fadrosh, 2019****).
4. Summarized technical replicates at the tree × time level to avoid artificially inflating sample size.

* Redford AJ, Bowers RM, Knight R, Linhart Y, Fierer N. The ecology of the phyllosphere: geographic and phylogenetic variability in the distribution of bacteria on tree leaves. Environ Microbiol. 2010 Nov;12(11):2885–93.

** Laforest-Lapointe I, Messier C, Kembel SW. Host species identity, site and time drive temperate tree phyllosphere bacterial community structure [Internet]. Vol. 4, Microbiome. 2016. Available from: <https://www.scopus.com/inward/record.uri?eid=2-s2.0-84995773049&doi=10.1186%2fs40168-016-0174-1&partnerID=40&md5=6ecd0c2c500af0e8c3d868235dfb4434>

*** Copeland JK, Yuan L, Layeghifard M, Wang PW, Guttman DS. Seasonal community succession of the phyllosphere microbiome. Vol. 28, Molecular Plant-Microbe Interactions. 2015. p. 274–85.

**** Ladau J, Eloe-Fadrosh EA. Spatial, Temporal, and Phylogenetic Scales of Microbial Ecology. Trends Microbiol. 2019 Aug;27(8):662–9.

Contamination controls in phyllosphere samples:

Low-biomass leaf-surface samples are prone to reagent contamination. Although negative controls are mentioned, the authors should present a short description or table showing the taxa detected in blanks, the number of ASVs removed, and evidence that blanks had negligible impact on the final community composition.

**Response:** Thank you for your thoughtful comment. In response, we have expanded the Methods section to provide a clearer description of the measures taken to minimize contamination during the processing of these low-biomass samples. Specifically, we now detail that all initial handling steps were performed inside a laminar-flow cabinet using new extraction kits and sterile, autoclaved materials to reduce the risk of reagent or environmental contamination.

MAG quality and pathway inference:

For metagenome-assembled genomes (MAGs) cited as carrying aromatic hydrocarbon degradation genes, provide completeness, contamination, and coverage values. Claims about “complete pathways” should rely only on MAGs with ≥70–80% completeness and <10% contamination. A supplementary table summarizing MAG metrics and associated KOs would improve transparency.

**Response:** Thank you for this helpful suggestion. We clarify that the metrics requested—completeness, contamination, and coverage values for all MAGs referenced in the manuscript—are provided in **S4 Table,** which reports the percentage of bin completeness and contamination for all samples (sampling period – urban sites) after bin refinement.

Functional inference limitations:

The conclusion that the phyllosphere “harbors robust genetic potential for xenobiotic degradation” should be moderated. The presence of catabolic genes indicates potential rather than confirmed activity. The authors should explicitly acknowledge this limitation and suggest follow-up functional validation (e.g., transcriptomics or enzyme assays).

**Response:** We fully agree. We have revised the Discussion and Conclusions to:

- Explicitly state that our results reflect **genetic potential,** not confirmed enzymatic or metabolic activity.
- Remove any phrasing that might imply direct functional expression.
- Suggest future **transcriptomic** or **enzyme-level** validation as important steps to confirm pathway activity.

Statistical transparency:

The diversity analyses (Shannon, Chao1, PERMANOVA) are appropriate but lack reporting of parameters such as the number of permutations, distance metric, and tests for dispersion (PERMDISP). Reporting these values and including R² effect sizes will enhance interpretability.

**Response:** We thank the reviewer for this valuable comment. We clarify that several of the statistical parameters mentioned—including the distance metric (Bray–Curtis) and the number of permutations used in PERMANOVA—were already present in the original manuscript, although not highlighted prominently. To improve transparency and ensure that all analyses are fully reproducible, we have now:

1. **Made these parameters more explicit in the revised Methods section**, including the distance metric and the exact number of permutations used.
2. **Added R² effect sizes** for all PERMANOVA models, which were part of the initial output but not previously reported.
3. **Included PERMDISP results**, which had already been performed as part of our beta-diversity workflow but were not previously described in the Methods nor reported in the Results. These results are now included to demonstrate that multivariate dispersion assumptions were met.
4. Provided **exact p-values** and ensured consistent reporting across all diversity analyses.

BTEX and pollution data alignment:

BTEX data were derived from 2022 monitoring, whereas sampling occurred in late 2023 and early 2024. The authors should clarify this temporal mismatch and discuss how it might influence interpretations linking pollutant levels to microbial gene content.

**Response:** Thank you for pointing out this temporal mismatch. We have now clarified in the revised manuscript that the 2022 BTEX monitoring campaign is the most recent dataset available from SIATA, the official environmental monitoring authority for the Aburrá Valley. Following the COVID-19 pandemic, SIATA restructured and prioritized other physicochemical and atmospheric monitoring programs, which resulted in the postponement of subsequent BTEX-specific campaigns. As a consequence, no BTEX measurements were conducted during 2023–2024.

We now explicitly state that the 2022 dataset serves as the closest available reference for characterizing site-level differences in exposure to aromatic hydrocarbons, but does not represent a time-matched measurement relative to our sampling period. The manuscript has been updated to emphasize that BTEX values were used solely as contextual indicators of the environmental profile of each site, and not as direct explanatory variables for microbial gene abundances.

Reproducibility and supplementary materials:

Include a detailed supplementary dataset showing per-sample sequencing metrics (raw reads, host-filtered reads, retained reads, number of scaffolds, MAG counts) and key gene/pathway completeness scores. This will ensure full reproducibility of the analyses.

**Response:** Thank you for this suggestion. In the revised manuscript, we have incorporated supplementary tables S1, S2, S3, and S5. These additions provide the full set of information required to reproduce our analyses.

Reviewer 1 — Minor Comments: Refined Responses

Abstract:

Indicate the number of samples (N = 12) and briefly mention sequencing platforms (MiSeq for 16S, NovaSeq for shotgun).

**Response:**Thank you for this helpful suggestion. We have revised the Abstract to explicitly report the sample size (N = 12) and to specify the sequencing platforms used (MiSeq for 16S amplicon sequencing and NovaSeq for shotgun metagenomics).

Phrase functional conclusions more cautiously, emphasizing “genetic potential.”

**Response:** We have also rephrased functional conclusions to emphasize that our findings reflect **genetic potential** for metabolic pathways, not confirmed metabolic activity.

Language and presentation:

The manuscript is well organized but needs minor English editing for grammar, tense consistency, and readability. Simplify complex sentences in the Abstract and Discussion and ensure consistent use of abbreviations (DT, SS, S1, S2).

**Response:** We appreciate this observation. The manuscript has undergone careful language revision to improve clarity, grammar, and readability. We simplified overly complex sentences, especially in the Abstract and Discussion, and standardized the use of all abbreviations across the text, tables, and figures.

Figures and tables:

Expand figure legends to define all abbreviations, sample sizes, and units.
Ensure table units (e.g., CO concentrations) are clearly stated and consistent.
Increase font size for axis labels and use colorblind-friendly palettes.

**Response:** Thank you for these important suggestions. We have expanded all figure legends to define abbreviations, sample sizes, and units; standardized units across all tables (including CO and other pollutants); and increased axis label sizes. We also updated color palettes to use **colorblind-friendly schemes** to improve accessibility.

Taxonomic naming:

Consider noting Pseudomonadota (Proteobacteria) at first mention for clarity to readers familiar with classical taxonomy.

**Response:** We agree and have added the dual nomenclature upon first mention (“Pseudomonadota (formerly Proteobacteria)” to ensure clarity for readers familiar with both taxonomic frameworks.

Ethical/collection statement:

Add a brief note confirming institutional or municipal permission for sampling plant material in Medellín.

**Response:** We have now clarified the permitting framework in the Methods. As described, sampling was conducted under the **General Permit for the Collection of Specimens of Wild Species of Biological Diversity for Non-Commercial Scientific Research**, issued by ANLA (Resolution 1566 of July 24, 2024). This permit covers sampling throughout Colombia, including the municipality of Medellín. A brief statement has been added to explicitly confirm compliance with local requirements.

Data availability statement:

The accession number (PRJNA1269120) should be cross-checked for functionality and included in the Data Availability section as a live link.

**Response:**

All code and data generated for this study have been deposited in a public repository in accordance with PLOS ONE’s data availability requirements. The dataset is available through the NCBI Sequence Read Archive under the following accession link: <https://www.ncbi.nlm.nih.gov/sra/PRJNA1269120>.

During the submission process, the NCBI platform required the specification of a public release date for the sequences, which was initially set to June 1, 2027. We have already contacted NCBI to request a modification of this date so that the data can be made publicly available as soon as possible. We will ensure that access is updated promptly and fully complies with the journal’s open data policies.

**Reviewer #2:**

The authors analyzed the bacterial phyllosphere community of Mangifera indica trees at two different sites, one with better air quality and one with poorer air quality. Sampling was conducted at two separate time points; however, it is unclear how many trees were sampled in total and whether the same trees were sampled at both time points.

Additionally, it is not specified whether the biological replicates refer to individual trees or composite samples from a single tree. If the latter, this would imply that only two trees were sampled—one at each location (if the same trees were sampled at both sampling times). This could mean that differences observed between sites are primarily due to microbiome variation between individual trees rather than true site-related differences. However, I assume that this is not the case. Anyway, clarification of the sampling strategy is needed.

**Response:**

**1. Number of trees and temporal sampling strategy**

Only one tree per site was sampled, and the same individuals were sampled at both time points. This design was intentionally chosen to examine *within-tree temporal dynamics* while minimizing confounding variation introduced by differences among host individuals—a factor known to strongly influence phyllosphere microbiome composition.

**2. Nature of the replicates (technical vs. biological)**

At each site and time point, the three leaf samples collected represent **technical replicates** derived from distinct positions within the *same* tree. These were processed independently to quantify local heterogeneity on a leaf-surface scale while ensuring that replicates were not treated as independent biological units.

**3. Rationale for this design**

Inter-individual host variability is one of the strongest drivers of leaf-surface microbial community structure. Several studies have demonstrated that even trees of the same species and located within the same site can harbor substantially different phyllosphere microbiomes. Therefore, sampling the *same* individual over time is a well-established approach for:

- Tracking temporal shifts with minimal confounding,
- Reducing high host-level variability that often obscures environmental signals,
- Preserving statistical interpretability in longitudinal microbiome analyses.

This logic is consistent with prior phyllosphere microbiome research (e.g., Redford et al., 2010*; Laforest-Lapointe et al., 2016**; Copeland et al., 2015***), where repeated sampling of single individuals has been used to examine environmental or seasonal influences.

**4. Statistical treatment to avoid pseudo-replication**

To ensure correct inference:

- Technical replicates were summarized per tree × time point and not treated as independent biological units.
- Longitudinal comparisons were modeled as repeated measures from the same tree.
- Cross-sectional comparisons between sites were interpreted cautiously and explicitly presented as differences *between two individuals representing two contrasting environments*, not as population-level effects.

This treatment aligns with best practices for non-independent microbiome sampling and avoids inflation of degrees of freedom.

**5. Clarifications added to the manuscript**

We have added explicit clarification in the Methods (Sampling Strategy, Statistical Analyses) stating:

- The number of trees sampled,
- The nature of the replicates,
- The justification for the design, and
- How technical replicates were handled to avoid pseudo-replication.

* Redford AJ, Bowers RM, Knight R, Linhart Y, Fierer N. The ecology of the phyllosphere: geographic and phylogenetic variability in the distribution of bacteria on tree leaves. Environ Microbiol. 2010 Nov;12(11):2885–93.

** Laforest-Lapointe I, Messier C, Kembel SW. Host species identity, site and time drive temperate tree phyllosphere bacterial community structure [Internet]. Vol. 4, Microbiome. 2016. Available from: <https://www.scopus.com/inward/record.uri?eid=2-s2.0-84995773049&doi=10.1186%2fs40168-016-0174-1&partnerID=40&md5=6ecd0c2c500af0e8c3d868235dfb4434>

*** Copeland JK, Yuan L, Layeghifard M, Wang PW, Guttman DS. Seasonal community succession of the phyllosphere microbiome. Vol. 28, Molecular Plant-Microbe Interactions. 2015. p. 274–85.

The authors report a lower abundance of xenobiotic biodegradation pathways at the more polluted sites, which appears counterintuitive. They do not comment on whether these differences are statistically significant. Currently, the only differences noted are in community structure between sites and higher alpha diversity at the more polluted sites.

**Response:**Thank you for this insightful observation. We agree that the initial presentation of the functional results required additional clarification. In the revised manuscript, we now explicitly address:

1.In this study, we did not perform gene expression analyses or quantitative assessments of functional gene abundance related to xenobiotic degradation pathways across sites. Consequently, conducting formal differential abundance or statistical significance testing for these pathways would be overly ambitious and could lead to misleading interpretations, as our data do not support such inferences.

Instead, our functional results are based on predicted metabolic potential inferred from ASV-level taxonomic assignments. As illustrated in Figure S3 (dot plot), this analysis highlights which metabolic pathways associated with the degradation and/or transformation of xenobiotic compounds are more frequently represented among ASVs detected at each site. These results are therefore intended to provide a qualitative and comparative overview of the potential functional repertoire associated with the microbial communities, rather than a quantitative or statistically tested comparison of pathway abundances.

2. Therefore, the reduced representation of xenobiotic degradation genes at the more polluted sites is ecologically plausible when interpreted as differences in genomic potential rather than gene expression or activity. Based on DNA-level analyses, our results indicate that the genetic repertoire required for complete catechol degradation is fully present in communities from less contaminated sites, whereas only partial pathway reconstruction was possible in communities from more polluted environments. Similar patterns have been reported in other low-biomass surface-associated microbiomes, where chronic environmental stress is associated with a loss or fragmentation of metabolically costly pathways (Simonsen, 2022*). We now discuss this point in the “Aromatic compound degradation pathways” section.

* Simonsen AK. Environmental stress leads to genome streamlining in a widely distributed species of soil bacteria. ISME J. 2022 Feb;16(2):423-434. doi: 10.1038/s41396-021-01082-x. Epub 2021 Aug 18. PMID: 34408268; PMCID: PMC8776746.

It would be beneficial to include analyses focusing on features that are exclusively present at the more polluted sites, maybe using a differential abundance analysis to identify potential marker taxa. Additionally, matching the MAGs with these marker taxa could facilitate the investigation of specific strains for interesting pathways and metabolic capabilities. Such findings could significantly enhance the study’s value.

**Response:**Thank you for this valuable suggestion. We agree that identifying site-associated taxa can strengthen ecological interpretation. However, differential abundance methods such as DESeq2 or similar count-based models require **independent biological replicates per condition** to avoid inflated false-positive rates and pseudo-replication. Because our study design includes **technical replicates from a single tree per site,** the statistical assumptions of these models cannot be met. This limitation is well documented in microbiome differential abundance literature (McMurdie & Holmes*, 2014; Weiss et al., 2017**).

Despite this, we incorporated aspects of the reviewer’s suggestion in ways that are statistically appropriate for our dataset. Specifically:

1. We now report **site-exclusive taxa and functional genes** using presence/absence contrasts, which do not rely on replication-based statistical models.
2. We provide **exploratory associations between MAGs and site-specific taxa**, without performing differential tests that are unsupported by the sampling structure.

* McMurdie, P. J., & Holmes, S. (2014). Waste not, want not: why rarefying microbiome data is inadmissible. PLOS Computational Biology, 10(4), e1003531.

** Weiss, S. et al. (2017). Normalization and microbial differential abundance strategies depend upon data characteristics. Microbiome, 5, 27.

While the metagenomic data have been deposited in the NCBI Sequence Read Archive, the raw amplicon data have not been made publicly available? Clarification is needed, otherwise the statement indicating that all data are freely accessible is inaccurate.

**Response:**

All code and data generated for this study have been deposited in a public repository in accordance with PLOS ONE’s data availability requirements. The dataset is available through the NCBI Sequence Read Archive under the following accession link: <https://www.ncbi.nlm.nih.gov/sra/PRJNA1269120>.

During the submission process, the NCBI platform required the specification of a public release date for the sequences, which was initially set to June 1, 2027. We have already contacted NCBI to request a modification of this date so that the data can be made publicly available as soon as possible. We will ensure that access is updated promptly and fully complies with the journal’s open data policies.

The manuscript's writing style is clear; however, the figures could be improved with more intuitive color schemes to facilitate interpretation. The paper would benefit from a better integration of the metagenomic and amplicon datasets to find novel results. Once all issue and comments (find more comments below) are resolved, I believe the paper is suitable for publication in PLOS ONE.

**Response:** We thank the reviewer for the observation. The suggested changes have been implemented. Specifically, the color palette was revised and consistently applied across all figures to improve clarity and accessibility. The selected palette is colorblind-friendly, following best practices for data visualization and in response to recommendations from another reviewer.

Comments:
Lines 180-182: If the negative controls are only used to check the dataset for ASVs exclusively present in the negative controls, then no contaminants are removed from the dataset at all. Maybe the negative controls can be shown on a PCOA plot together with the other samples. If they do not cluster with the other samples, then there should be no problem.

**Response:** Thank you for your thoughtful comment. In response, we have expanded the Methods section to provide a clearer description of the measures taken to minimize contamination during the processing of these low-biomass samples. Specifically, we now detail that all initial handling steps were performed inside a laminar-flow cabinet using new extraction kits and sterile, autoclaved materials to reduce the risk of reagent or environmental contamination. Because no negative control libraries were generated, it is not possible or methodologically appropriate to include controls in beta-diversity analyses.

We have clarified this explicitly in the Methods section to ensure transparency regarding the scope and limitations of contamination assessment in this study. Importantly, all downstream community-level analyses are based solely on biological samples processed and sequenced under identical conditions, and the reported beta-diversity patterns reflect differences among these samples rather than artifacts introduced by control comparisons.

Lines 118-136: I am not sure if I understood the sampling strategy correctly. More details would be useful. For example, it is written that 12 samples were taken. Does this refer to 12 different trees? Were the same trees sampled again at S2 or different trees? Is one biological replicate one tree? Or were multiple composite samples taken from one tree?

**Response:** We thank the reviewer for this observation. The points raised here were addressed in response to a previous reviewer’s comment. In addition, we have further clarified and expanded the sampling methodology in the corresponding section of the manuscript to improve transparency and reproducibility.

Lines 325-326: “both of which include genera known for their roles in organic pollutant degradation.” This statement needs a reference.

**Response:** Thank you for your thoughtful comment. We now include the reference in the text.

Lines 337-338: The authors refer here to Fig 1e-f and 1f is showing the species, but the text is only focusing on the genera, which is a bit misleading. I would suggest to either remove species (1f) from the figure or also mention the species in the text. It would anyway be interesting to talk about the species shown in 1f since there is also the Flavobacteriales endosymbiont, indicating that a significant proportion of the community are insect endosymbionts? I think this is a point for the discussion.

**Response: We thank the reviewer for this comment. we have revised the text to include representative species-level assignments shown in Fig. 1f (e.g., Sphingomonas sp., Massilia sp., and Pantoea agglomerans). In addition, we now explicitly mention the detection of a Flavobacteriales endosymbiont and briefly discuss this finding as a potential indication of insect-mediated contributions to the phyllosphere microbiome.**

Line 331: Figure 1: In general, I am wondering what exactly is shown in figure 1. I cannot believe that the whole bacterial community are only 10 different genera or 10 different species. In your alpha diversity plots we see an estimated chao richness of 1000 up to 3000 different ASVs…. Are only taxa shown above a certain abundance threshold? Why not summarize the rest in “Other” and give an impression about the whole bacterial community? Please revise Figure 1 or at least state in the figure legend what is shown. Otherwise, the figure is quite misleading telling us that there are only 10 different species in the phyllosphere of Mangifera indica.

**Response:** Thank you for your thoughtful comment. The suggested adjustment has been made.

Line 351: Figure 2: In my opinion, figure 2 would be easier to read if different color schemes are used depending what is compared. I would suggest to use different colors for the sites and the sampling times, and not to use the same colors for sites and sampling times.

**Response:** We thank the reviewer for the observation. The suggested changes have been implemented. Specifically, the color palette was revised and consistently applied across all figures to improve clarity and accessibility. The selected palette is colorblind-friendly, following best practices for data visualization and in response to recommendations from another reviewer.

While site-level differences were initially considered a central hypothesis, the statistical analyses revealed that temporal variation had a stronger effect on community structure than spatial differences among sites. For this reason, the color scheme in Figure 3 was intentionally designed to highlight sampling times rather than sites, as this representation more accurately reflects the primary driver of variation supported by the data.

Line 374: Figure 3: Since the two different colors are used for the sampling times, the focus of the figure is more on the sampling times and not on the sites. Not sure if this is intended. I would suggest to use the different colors to differentiate between the sites, which is the focus of the whole story.

**Response:** We thank the reviewer for this observation. We already explained the reason for this design in a previous comment.

Line 417: Figure 4: In my opinion, the coloring and the order of the bars is misleading. Again, the focus of this figure seems to be on the comparison of sampling time 1 against sampling time 2. I would suggest to change the order to DT S1, DT S2, SS S1, SS S2. If there are significant differences, I would suggest to indicate that in the figure.

**Response:** We thank the reviewer for the observation. We already explained the reason for this design in a previous comment.

Lines 423-429: I am not sure if the statements made here are correct. It is written that the presence of xenobiotics degradation pathways supports the hypothesis of phyllosphere bacteria being equipped with these pathways. Well yes, but would this not mean that in the more polluted environment, the abundance of the xenobiotics degradation pathways should be higher, not the other way around as suggested in this work? The abundance of xenobiotics degradation pathways is clearly less abundant in the more polluted DT S1 compared to the less polluted SS S1, as shown in figure 4B.

**Response:** We thank the reviewer for the observation. We already explained this in a previous comment.

Line 455: Mangifera indica not italic

**Response:** Thank you for your thoughtful comment. The suggested adjustment has been made.

Reviewer #3:

The study reported the taxonomic and functional composition of Mangifera indica, a species of phyllosphere bacteria, and their relationship with urban air pollution in an urban tropical location, Medellín, Colombia. The authors collected samples from tree leaves (n = 12) in two seasons at two urban sites to conduct an analysis of the microbial communities using multi-omics approaches (i.e., 16S rRNA amplicon sequencing and shotgun metagenomics). The authors found taxonomically diverse communities that harbor complete pathways for degrading airborne pollutants at two sites, and functional diversity was shaped by both spatial and temporal factors. Overall, the methods are validated, and the findings have implications for bioremediating urban air pollution. However, some issues need to be addressed before considering publication in PLOS One. My specific comments are as follows:

1. Line 80: Define the acronyms BTEX and PAHs at their first occurrence.
2. Line 243: Define the acronym FDR at its first occurrence.
3. Lines 254–309: Subscript the numbers for the terms of air pollutants, including PM2.5, PM10, NO2, and O3; report corresponding standard deviations for their average levels; and perform statistical difference tests for their levels between seasons and sites to support the site-specific arguments.
4. Results writing style: Present the results logically instead of in a figure-by-figure manner.
5. Line 402: Where is section 3.4?
6. Color scheme for Figure 1: Use different color schemes for different taxonomic levels.

**Response:** We thank the reviewer for the observations. We thank the reviewer for the detailed and constructive comments. All suggested revisions have been carefully addressed in the revised manuscript. Specifically, all acronyms (BTEX, PAHs, and FDR) are now defined at their first occurrence; chemical formulas and pollutant names (PM₂.₅, PM₁₀, NO₂, and O₃) have been properly formatted with subscripts; It was not possible to calculate and include the standard deviation in the pollutant levels, as it was provided by a state entity.

The Results section has been reorganized to improve logical flow and readability. The missing subsection numbering has been corrected, and the color scheme of Figure 1 has been revised using a colorblind-friendly palette with a limited number of distinct colors to ensure clear differentiation among taxonomic levels while maintaining a consistent visual scheme across all panels.

Reviewer #4: This study investigates how urban pollution gradients influence the taxonomic and functional composition of Mangifera indica phyllosphere bacterial communities. Overall, this is a nice study and a well-organized draft, with a clear structure, detailed methodological description, and accurate presentation of results; however, a few minor issues remain that should be addressed before publication.

1. The introduction is informative and well structured, but it is overly detailed, particularly in the global and regional background sections. The extensive statistics and descriptive information make the main research question less focused. This section would benefit from condensing the general context, improving transitions between ideas, and more clearly emphasizing the specific knowledge gap and study objectives. Overall, a shorter and more targeted introduction would enhance both readability and impact.
A similar issue of excessive detail is also present in the Methods section. I recommend reviewing the paper (PMID: 35094961) as an example of how to describe analytical procedures clearly and concisely.

**Response:** We thank the reviewer for the thoughtful comments. The Introduction was only minimally revised, as other reviewers considered this section to be clear, well structured, and appropriate in scope, and we aimed to maintain consistency with those evaluations. Minor edits were made to improve clarity without substantially altering its length or focus.

In contrast, the Methods section was expanded, particularly the sampling subsection, in direct response to requests from other reviewers seeking greater methodological detail to ensure transparency and reproducibility. We therefore prioritized these revisions while maintaining a balance between conciseness and completeness across sections.

2. In the section “Taxonomic characterization of phyllosphere bacteria,” please clarify whether the removal of low-abundance ASVs was performed before or after rarefaction, as the current description leaves the sequence of procedures unclear.

**Response:** Thank you for your thoughtful comment. The suggested adjustment has been made.

3. For Tables 1 and 2, please consider visualizing the data as line charts, adding WHO standard reference lines to improve readability and facilitate comparison.

**Response**: We thank the reviewer for this helpful suggestion. While visualizing the data as line charts with WHO reference values can indeed improve readability in many contexts, we chose not to include WHO standard reference lines in this case to avoid potential misinterpretation. WHO air quality guideline values are defined based on daily or, in some cases, multi-day or annual averaging periods, whereas the data used in this study were provided by the responsible monitoring agency (SIATA) as point measurements corresponding to specific sampling times.

Because our dataset does not include the temporal resolution required to compute daily or weekly averages, directly overlaying WHO guideline thresholds on these point measurements could be misleading and imply a level of comparability that is not supported by the data.

4. In Figure 1f, all taxa are shown at the species level and sum to 1. However, the sequencing method used in this study—the 16S rRNA V3–V4 region—generally does not provide sufficient resolution for reliable species-level identification. Please clarify how these taxa were assigned to the species level.

**Response:**W e thank the reviewer for this comment. While we acknowledge that the 16S rRNA V3–V4 region does not universally resolve all taxa at the species level, in our dataset several ASVs could be assigned to species with sufficient confidence based on sequence similarity to reference databases. For this reason, species-level taxa were included in Fig. 1f and only the most abundant assignments are shown. As indicated in the figure legend, Fig. 1f displays the ten most abundant taxa at the species level, which were normalized to facilitate visual comparison; consequently, their relative abundances sum to one.


5. There is some redundancy in describing data processing steps in both the Methods and Results sections. Since these procedures are already detailed in the Methods, consider streamlining the Results section to focus more directly on the findings rather than methodological repetition.

**Response:** Thank you for your thoughtful comment. The suggested adjustment has been made.

6. PLOS authors have the option to publish the peer review history of their article ([what does this mean?](https://track.editorialmanager.com/CL0/https:%2F%2Fjournals.plos.org%2Fplosone%2Fs%2Feditorial-and-peer-review-process%23loc-peer-review-history/1/010f019a32d6a5cc-3f3b2389-07e1-4830-b973-caa9fc88b0c5-000000/0ZksKRE-bM-KYlqYQb2mj5kh24k-zugxLgN9hm-6EdU=235)). If published, this will include your full peer review and any attached files. If you choose “no”, your identity will remain anonymous but your review may still be made public.

**Do you want your identity to be public for this peer review?** For information about this choice, including consent withdrawal, please see our [Privacy Policy](https://track.editorialmanager.com/CL0/https:%2F%2Fwww.plos.org%2Fprivacy-policy/1/010f019a32d6a5cc-3f3b2389-07e1-4830-b973-caa9fc88b0c5-000000/o4-9GUQbxEfwYiqfy8VW0zi0TlvWdWO7hdfC4AB4G94=235).

Reviewer #1: No

Reviewer #2: No

Reviewer #3: No

Reviewer #4: No
