# Supplementary material for: Metagenomic and taxonomic profiling of phyllosphere bacteria from Mangifera indica in response to urban air pollutants in Medellín, Colombia
Source: PLoS One. 2026 Apr 28;21(4):e0347959. doi: 10.1371/journal.pone.0347959 (PMC13124002; doi:10.1371/journal.pone.0347959)
Supplement: Attachment — Submitted filename: Response_to_Reviewers_PLOSONE_19.03.2026.docx [file pone.0347959.s003.docx]

**Response to Journal Requirements — PLOS ONE**

**Manuscript ID:** PONE-D-25-50660R1

**Title:** *Metagenomic and taxonomic profiling of phyllosphere bacteria from Mangifera indica in response to urban air pollutants in Medellín, Colombia.*

Dear Academic Editor,

We thank you for the opportunity to revise our manuscript and for the detailed guidance provided. Below, we address each journal requirement point-by-point and describe the corresponding actions taken in the revised submission.

**Comments to the Author**

1. If the authors have adequately addressed your comments raised in a previous round of review and you feel that this manuscript is now acceptable for publication, you may indicate that here to bypass the “Comments to the Author” section, enter your conflict of interest statement in the “Confidential to Editor” section, and submit your "Accept" recommendation.

Reviewer #1: All comments have been addressed

Reviewer #2: (No Response)

Reviewer #3: All comments have been addressed

Reviewer #4: All comments have been addressed

2. Is the manuscript technically sound, and do the data support the conclusions?

The manuscript must describe a technically sound piece of scientific research with data that supports the conclusions. Experiments must have been conducted rigorously, with appropriate controls, replication, and sample sizes. The conclusions must be drawn appropriately based on the data presented.

Reviewer #1: Yes

Reviewer #2: No

Reviewer #3: Yes

Reviewer #4: Yes

3. Has the statistical analysis been performed appropriately and rigorously?

Reviewer #1: Yes

Reviewer #2: No

Reviewer #3: Yes

Reviewer #4: Yes

4. Have the authors made all data underlying the findings in their manuscript fully available?

The PLOS Data policy requires authors to make all data underlying the findings described in their manuscript fully available without restriction, with rare exception (please refer to the Data Availability Statement in the manuscript PDF file). The data should be provided as part of the manuscript or its supporting information, or deposited to a public repository. For example, in addition to summary statistics, the data points behind means, medians and variance measures should be available. If there are restrictions on publicly sharing data—e.g. participant privacy or use of data from a third party—those must be specified.

Reviewer #1: Yes

Reviewer #2: Yes

Reviewer #3: Yes

Reviewer #4: Yes

5. Is the manuscript presented in an intelligible fashion and written in standard English?

PLOS ONE does not copyedit accepted manuscripts, so the language in submitted articles must be clear, correct, and unambiguous. Any typographical or grammatical errors should be corrected at revision, so please note any specific errors here.

Reviewer #1: Yes

Reviewer #2: Yes

Reviewer #3: Yes

Reviewer #4: Yes

6. Review Comments to the Author

Please use the space provided to explain your answers to the questions above. You may also include additional comments for the author, including concerns about dual publication, research ethics, or publication ethics. (Please upload your review as an attachment if it exceeds 20,000 characters)

Reviewer #1: (No Response)

Reviewer #2: Dear authors,

Thank you for addressing all of my comments. Unfortunately, however, with sampling only one individual tree per site, it is impossible to say if the pollution is the driver of the observed differences in the phyllosphere microbiome, or if the differences are primarily due to microbiome variation between individual trees.

You mentioned the following publication, in which a similar sampling strategy was used according to you: Laforest-Lapointe I, Messier C, Kembel SW. Host species identity, site and time drive temperate tree phyllosphere bacterial community structure.

I am familiar with that study. In their research, Laforest-Lapointe et al. sampled three times during the 2013 growing season (June, July, and August) from three individual trees per species at each site, totaling 180 samples. They also found that individual trees harbored distinct microbial communities.

You mention in your comments to the reviewer:

Only one tree per site was sampled, and the same individuals were sampled at both time points. This design was intentionally chosen to examine within-tree temporal dynamics while minimizing confounding variation introduced by differences among host individuals—a factor known to strongly influence phyllosphere microbiome composition.

However, within-tree temporal dynamics are not shown in the manuscript. Which, again, is hard to do with only to sampling timepoints.

It may be worthwhile to examine whether individual amplicon sequence variants (ASVs) show any response to particulate matter (PM) levels, potentially through Spearman correlations or similar statistical approaches. Identifying strains that exhibit significant associations with PM values could help determine if these strains may be driving differences in the genetic repertoire related to xenobiotic degradation.

Further, you could consider splitting the dataset by individual trees to investigate whether temporal changes in the microbiome occur within each tree. Comparing these changes across trees can help determine if the microbiome’s temporal dynamics are consistent, especially given that pollution levels increased at both locations between sampling points. Nonetheless, caution should be exercised, as observed changes may be influenced by seasonal variations rather than air pollution alone.

Given the current manuscript, it is difficult to convincingly conclude that observed microbiome changes are caused by air pollution rather than inter-individual differences or seasonal effects.

**Response:**

We thank the reviewer for this thoughtful and detailed assessment. We fully acknowledge that sampling a single tree per site limits the ability to disentangle pollution-driven effects from inter-individual variability. However, we would like to further clarify that this sampling strategy was intentionally designed to control for a major and well-documented source of variability in phyllosphere microbiome studies: host genetic heterogeneity.

In the case of *Mangifera indica*, urban plantings—particularly in tropical cities such as Medellín—are typically composed of multiple cultivars and genotypes, often propagated through grafting or seed, resulting in substantial intra-specific genetic diversity. This genetic variability has been shown to influence plant-associated microbial communities by modulating leaf physicochemical traits and microenvironmental conditions.

To avoid introducing this additional layer of uncontrolled variation, we deliberately sampled the same individual tree at each site across both time points. This approach is consistent with recommendations in phyllosphere ecology, where repeated sampling of the same host individual is used to isolate temporal dynamics while minimizing host-driven confounding effects.

Supporting this, studies on mango genetic diversity in similar regions have documented significant intra-specific variability. For example:

- Viruel, M. A., Escribano, P., Barbieri, M., Ferri, M., & Hormaza, J. I. (2005). Fingerprinting, embryo type and geographic differentiation in mango (*Mangifera indica* L., Anacardiaceae) with microsatellites. *Molecular Breeding, 15*(4), 383–393. https://doi.org/10.1007/s11032-004-7982-x
- Ravishankar, K. V., Dinesh, M. R., Nischita, P., & Sandya, B. S. (2015). Development and characterization of microsatellite markers in mango (*Mangifera indica*) using next-generation sequencing technology and their transferability across species. *Molecular Breeding, 35*, Article 93. <https://doi.org/10.1007/s11032-015-0289-2>
- Yan, J., Zheng, B., Wang, S., Xu, W., Qian, M., Ma, X., & Wu, H. (2024). Genetic diversity and fingerprinting of 231 mango germplasm using genome SSR markers. *International Journal of Molecular Sciences, 25*(24), 13625. https://doi.org/10.3390/ijms252413625
- Liang, Q., Pan, H., He, X., Wang, S., Hou, Y., Xiao, H., Xu, G., Yi, R., Lin, D., & Yang, Z. (2024). Population structure and genetic diversity of mango (*Mangifera indica* L.) germplasm resources as revealed by single-nucleotide polymorphism markers. *Frontiers in Plant Science, 15*, 1328126. https://doi.org/10.3389/fpls.2024.1328126

These studies demonstrate that mango populations can harbor high levels of genetic diversity, which in turn may influence associated microbiomes.

We would like to clarify the conceptual scope of our study. The main objective was to characterize the taxonomic composition and functional potential of the phyllosphere bacterial community at the community level, rather than to evaluate responses of individual ASVs or strains. To achieve this, we combined 16S rRNA metabarcoding and shotgun metagenomics to analyze the collective genetic repertoire associated with the degradation of atmospheric aromatic hydrocarbons, particularly compounds from the BTEX group. Consequently, the functional analysis focuses on metabolic pathways related to the degradation of these volatile organic compounds, rather than on pollutants associated with particulate matter. We consider that this study provides a baseline characterization of the phyllosphere microbiome and its functional potential in a tropical urban environment, which may guide future research focusing on strain-level microbial responses or on the identification of specific microbial bioindicators of pollution.

Importantly, our results indicate that temporal variation was the main factor structuring the microbial community. Beta diversity analysis based on Bray–Curtis distances showed a significant separation between sampling periods (PERMANOVA, p = 0.005; R² = 0.7403), while spatial differences between sites had a smaller effect. In the PCoA ordination, the first axis, which explained the largest proportion of variance, was primarily associated with the sampling period, indicating that changes in environmental conditions between December 2023 and April 2024—coinciding with a documented air pollution episode in the Aburrá Valley—were strongly associated with shifts in microbial community composition. These results support the interpretation that temporal environmental variation, including seasonal dynamics of air pollution, exerted a stronger influence on community structure than spatial differences between sampling sites.

Reviewer #3: (No Response)

Reviewer #4: Thanks to the authors for their hard work. All my questions have been properly addressed and explained.

7. PLOS authors have the option to publish the peer review history of their article (what does this mean?). If published, this will include your full peer review and any attached files.

If you choose “no”, your identity will remain anonymous but your review may still be made public.

Do you want your identity to be public for this peer review? For information about this choice, including consent withdrawal, please see our Privacy Policy.

Reviewer #1: No

Reviewer #2: No

Reviewer #3: No

Reviewer #4: No

[NOTE: If reviewer comments were submitted as an attachment file, they will be attached to this email and accessible via the submission site. Please log into your account, locate the manuscript record, and check for the action link "View Attachments". If this link does not appear, there are no attachment files.]

To ensure your figures meet our technical requirements, please review our figure guidelines: https://journals.plos.org/plosone/s/figures

You may also use PLOS’s free figure tool, NAAS, to help you prepare publication quality figures: https://journals.plos.org/plosone/s/figures#loc-tools-for-figure-preparation.

NAAS will assess whether your figures meet our technical requirements by comparing each figure against our figure specifications.
